# Genome-Wide Identification of *FCS-Like Zinc Finger (FLZ)* Family Genes in Three *Brassica* Plant Species and Functional Characterization of *BolFLZ*s in Chinese Kale Under Abiotic Stresses

**DOI:** 10.3390/ijms252312907

**Published:** 2024-11-30

**Authors:** Yuwan Zhao, Shunquan Chen, Mao Qin, Kejuan Shui, Riqing Li, Baoli Yang, Jin Liu, Zhufeng Chen

**Affiliations:** 1 Center for Biological Science and Technology, Key Laboratory of Cell Proliferation and Regulation Biology of Ministry of Education, Zhuhai Macao Biotechnology Joint Laboratory, Faculty of Arts and Sciences, Beijing Normal University, Zhuhai 519087, China; ywanzhao@163.com; 2 Shenzhen Inspection and Testing Center of Agricultural Product Quality and Safety, Shenzhen 518071, China; 2018010158@m.scnu.edu.cn (S.C.); mqin@outlook.com (M.Q.); liriqing246@163.com (R.L.); ybl0212@163.com (B.Y.); ahappyheart@126.com (J.L.); 3 Technical Center of Gongbei Customs, Zhuhai 519087, China; yishuhuaiciwater@163.com

**Keywords:** abiotic stresses, *Brassica*, Chinese kale, expression analysis, *FLZ* gene family

## Abstract

FCS-like zinc finger (FLZ) proteins are plant-specific regulatory proteins, which contain a highly conserved FLZ domain, and they play critical roles in plant growth and stress responses. Although the *FLZ* family has been systematically characterized in certain plants, it remains underexplored in *Brassica* species, which are vital sources of vegetables, edible oils, and condiments for human consumption and are highly sensitive to various abiotic stresses. Following the whole-genome triplication events (WGT) in *Brassica*, elucidating how the *FLZ* genes have expanded, differentiated, and responded to abiotic stresses is valuable for uncovering the genetic basis and functionality of these genes. In this study, we identified a total of 113 *FLZ* genes from three diploid *Brassica* species and classified them into four groups on the basis of their amino acid sequences. Additionally, we identified 109 collinear gene pairs across these *Brassica* species, which are dispersed among different chromosomes, suggesting that whole-genome duplication (WGD) has significantly contributed to the expansion of the *FLZ* family. Subcellular localization revealed that six representative BolFLZ proteins are located in the nucleus and cytoplasm. Yeast two-hybrid assays revealed that 13 selected BolFLZs interact with BolSnRK1α1 and BolSnRK1α2, confirming the conservation of the SnRK1α-FLZ module in *Brassica* species. Expression profile analysis revealed differential expression patterns of *BolFLZ* across various tissues. Notably, the expression levels of seven *BolFLZ* genes out of the fifteen genes analyzed changed significantly following treatment with various abiotic stressors, indicating that the *BolFLZ* genes play distinct physiological roles and respond uniquely to abiotic stresses in *Brassica* species. Together, our results provide a comprehensive overview of the *FLZ* gene family in *Brassica* species and insights into their potential applications for enhancing stress tolerance and growth in Chinese kale.

## 1. Introduction

FCS-like zinc finger (FLZ) proteins represent a class of plant-specific regulatory proteins characterized by a conserved FLZ domain, also known as domain of unknown function 581 (DUF581) [1]. The FLZ domain comprises an assembly of 70 amino acid residues possessing a conserved CX2CX17–19FCSX2C motif [2]. The first protein harboring the FLZ domain was identified through aging-associated enhancer capture experiments [3]. This protein can be induced by senescence and is crucial in abscisic acid (ABA)-mediated seed dormancy, which is a desirable trait in agricultural production because it inhibits the preharvest sprouting of many grains and seeds [4]. Since the importance of FLZ proteins in plant stress resistance and developmental growth has been progressively revealed, plant scientists have increasingly focused their attention on extensive investigations of the *FLZ* gene family, aiming to increase crop resilience and yield under various environmental conditions.

To date, members of the *FLZ* gene family have been shown to be essential for various biological processes, including plant development, senescence, stress responses, and sugar signaling [4,5,6,7]. Recent research has indicated that the *FLZ* gene is a critical component of the gene regulatory network involved in controlling flowering time in plants. In Arabidopsis, AtFLZ13 negatively regulates flowering time by promoting FLOWERING LOCUS C (FLC) transcription with ABI5 and interacting with FLC to inhibit FLOWERING LOCUS T (FT) and SUPPRESSOR OF OVEREXPRESSION OF CONSTANS 1 (SOC1) [8]. Additionally, OsFLZ2 interacts with OsMADS51, a rice orthologue of Arabidopsis FLC, to finely tune flowering time under short-day conditions [9,10]. These findings suggest that FLZ proteins could function as transcription factors to regulate gene expression. In addition to those in rice and Arabidopsis, several studies across diverse plant species, including maize (*Zea mays*), wheat (*Triticum aestivum*), tomato *(Solanum lycopersicum)*, cotton (*Gossypium hirsutum*), and liquorice (*Glycyrrhiza inflata*), have revealed that FLZ proteins play significant roles in mediating responses to various stress conditions [5,6,7,11,12]. For example, *ZmFLZ14* negatively regulates a plant’s response to carbon starvation [13], whereas certain *GhFLZ* genes serve as key regulatory factors in the response of *G. hirsutum* to cold stress [12].

Most notably, FLZ proteins serve as scaffolding proteins for sucrose nonfermenting related kinase 1 (SnRK1), interacting with its kinase subunits to assist SnRK1 in sensing energy deficits caused by adverse conditions and coordinating cellular responses for adaptation in plants [1,14,15,16]. SnRK1 acts as a central integrator of energy metabolism, stress, and developmental signals [14,17]. SnRK1 promotes catabolism while minimizing anabolism by reprogramming the transcriptional machinery, thus enabling plants to survive under low-energy conditions and increasing tolerance to various biotic and abiotic stresses by regulating the transcription of genes associated with stress tolerance [17,18,19]. In Arabidopsis, AtFLZ3 (DUF581-9) functions as a novel negative regulator of the key energy sensor SnRK1 by inhibiting its activity through the physical blockade of the phosphorylation of its catalytic subunit KIN10 under energy-sufficient conditions. Under low-energy conditions, AtFLZ3 undergoes partial degradation by the proteasome, alleviating its inhibition and allowing the plant to perform essential low-energy responses [20,21]. In rice, a yeast two-hybrid assay revealed strong interactions between eight OsFLZ proteins and SnRK1A, with OsFLZ18 negatively regulating SnRK1 activity to suppress αAmy3 expression, which subsequently modulates rice seed germination and coleoptile growth under submerged conditions [22]. Given the profound impact of *FLZ*s on plant development regulation and their critical role in coordinating responses to biotic and abiotic stress signals, identification and functional studies of plant *FLZ* family genes have attracted increasing attention. However, the genome-wide identification and annotation of *FLZ* genes have yet to be reported in *Brassica* species. Novel insights into FLZ proteins from a diverse array of plant species, particularly those within horticultural crops, are essential.

*Brassica* species, which are rich in vitamins, minerals, and phytochemicals that offer numerous health benefits, serve as a global source of essential nutrients and edible vegetable oils for human consumption [23,24]. A significant whole-genome triplication (WGT) event occurred in almost all commercially important *Brassica* species approximately 22.5 million years ago, resulting in three diploid species (*Brassica rapa*, AA, 2n = 20; *B. nigra*, BB, 2n = 16; and *B. oleracea*, CC, 2n = 18) and three allotetraploid species (*B. juncea*, AABB, 2n = 36; *B. napus*, AACC, 2n = 38; and *B. carinata*, BBCC, 2n = 34) [25,26]. The evolutionary relationships among the six crop species are depicted by U’s triangle, wherein the three key diploids form the vertices, whereas the three allotetraploids originated from interspecific hybridization between the diploids [27]. Whole-genome sequencing of *Brassica* species has demonstrated that whole-genome triplication (WGT) not only plays an important role in the speciation and morphotype diversification of these plants but also contributes to the diversification of stress-related genes in *Brassica* crop species [26,28]. In *Brassica rapa*, genes that respond to environmental stresses were preferentially retained after WGT, which is regarded as a mechanism for protecting plants from harmful stresses [29]. As a result of WGD, WGT, and tandem and segmental duplications, the number of members within numerous multigene families has expanded across various *Brassica* crop species, exhibiting responsiveness to a range of diverse stresses [28]. Approximately 3% and 4% of tandemly duplicated genes are responsive to abiotic stresses in *B. rapa* and *B. oleracea*, respectively [30]. Moreover, because of WGD, numerous genes have been identified in *Brassica* species in response to salt stress [31]. Given the considerable variability in stress sensitivity among *Brassica* species and varieties [28,32], studying stress-regulated genes in *Brassica* provides insights into gene function and evolution while also expanding opportunities for genetic engineering of crops resistant to diverse stresses.

In this study, we hypothesize that if the *FLZ* gene family in *Brassica* species has expanded through WGT events, there will be an increased number of genes and gene differentiation, which may lead to each exhibiting unique responses to abiotic stresses within these species. To test this hypothesis, we performed a comprehensive genome-wide analysis of the *FLZ* gene family across three *Brassica* plant species, examining chromosomal distribution, conserved amino acid residues in the FLZ-type domain, phylogenetic relationships, synteny analysis, gene structure, conserved motifs, and potential cis-acting elements in the promoter. Additionally, we investigated the subcellular localization and spatiotemporal expression patterns of BolFLZ proteins from Chinese kale (*Brassica oleracea* L. var. *alboglabra*), an original Chinese *Brassica* vegetable known for its high nutritional value [33]. We also examined the potential protein–protein interactions between BolFLZs and BolSnRK1α, as they form a complex associated with diverse functions. Finally, we conducted a detailed analysis of the responsiveness of the *BolFLZ* gene family to various stresses in Chinese kale via quantitative real-time PCR (qRT–PCR). This research provides new insights into FLZ proteins across *Brassica* species, highlighting their potential role in the response to abiotic stress. The findings of this study provide valuable reference data and a theoretical foundation for future research on the biological functions of the *FLZ* gene family in *Brassica*.

## 2. Results

### 2.1. Identification and Classification of FLZ Genes in the Three Brassica Plant Species

In this study, BLAST was initially carried out to search for *FLZ* genes in the genomes of three *Brassica* species. The obtained sequences were further verified through a hidden Markov model via previously described methods. Finally, we identified 36, 41, and 36 *FLZ* genes in Chinese cabbage (*Brassica rapa*; Bca), black mustard (*Brassica nigra*; Bni), and Chinese kale (*Brassica oleracea*; Bol), respectively. These genes were designated *BcaFLZ1-BcaFLZ36*, *BniFLZ1-BniFLZ41*, and *BolFLZ1-BolFLZ36* on the basis of their chromosomal locations (Table S1). These findings revealed that the number of *FLZs* slightly varied between species, which could be attributed to the loss or duplication of genes throughout the evolutionary history of *Brassica*.

Detailed information for each *FLZ* gene, including the gene name, locus ID, chromosome location, open reading frame (ORF), protein length, predicted molecular weight (MW), isoelectric point (pI), and subcellular localization, is presented in Table S1. Among the 113 putative FLZ proteins, the lengths of the protein sequences encoded by the FLZ genes vary from 69 amino acids in BniFLZ19 to 335 amino acids in BcaFLZ14, and the relative molecular weights range from 8.018 to 37.322 kDa. The theoretical pI values range from 4.56 for BcaFLZ35 to 10.03 for BolFLZ5, with 80 proteins having a pI value greater than 7 and the remaining proteins having a pI value less than 7, suggesting that this gene family is biased towards basic amino acids. The aliphatic indices of these 113 putative FLZ proteins ranged from 38.57 to 90.48. The grand average of hydropathicity (GRAVY) scores, which are all less than zero, range from −1.458 for BniFLZ25 to −0.245 for BcaFLZ14, suggesting that these proteins are hydrophilic. Considering that proteins with an instability index less than 40 are classified as stable, all FLZ proteins in the three *Brassica* species are predicted to exhibit instability coefficients exceeding 40, which indicates significant instability. These results confirm that the 113 putative FLZ proteins across the three *Brassica* plant species exhibit significant variation in their protein characteristics.

### 2.2. Phylogenetic Analysis of FLZ Proteins

**Figure 1:**
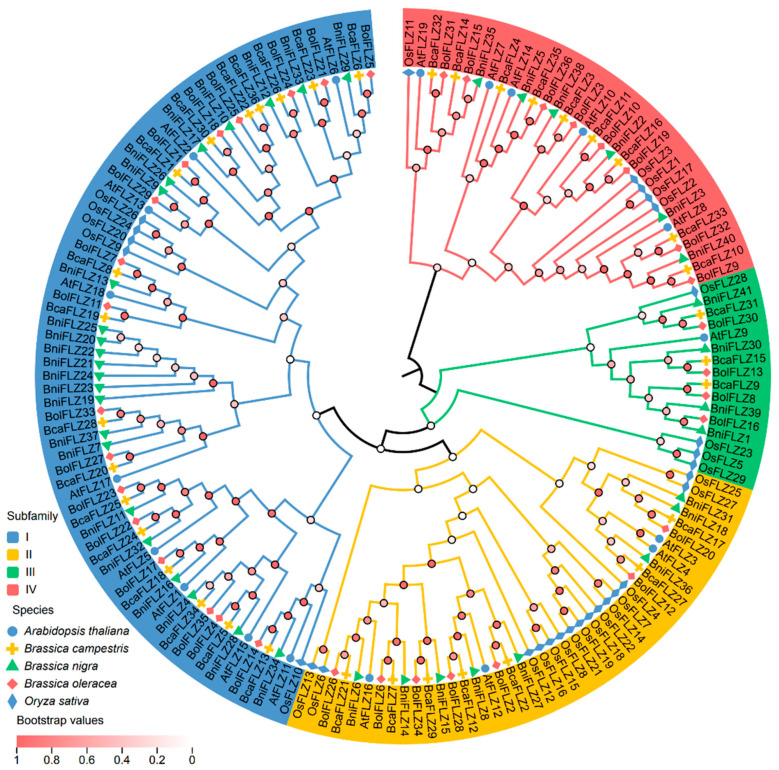
Phylogenetic tree of the FLZs in the three *Brassica* species, Arabidopsis, and rice. The phylogenetic tree was generated via the neighbor-joining (NJ) method implemented in MEGA X software (version 11.0) with the JTT model and pairwise gap deletion option. Bootstrap analysis was conducted with 1000 iterations, and the bootstrap values are represented by the color intensity of the circles at each node in the phylogenetic tree. The phylogenetic tree is divided into four subgroups, represented by blue, yellow, red, and green. The FLZ proteins from the five species are denoted with distinct shapes and colors in the phylogenetic analysis.

To investigate the phylogenetic relationships among FLZ proteins, the amino acid sequences of FLZs from three *Brassica* species (*Brassica rapa*, *Brassica nigra*, and *Brassica oleracea*), rice (*O. sativa*), and Arabidopsis (*A. thaliana*) were utilized to construct an unrooted phylogenetic tree via the neighbor-joining method (Figure 1). The analysis categorized these proteins into four monophyletic clades on the basis of sequence identity. Clade I, the largest, comprises 71 FLZ members, followed by Clade II, with 41 members, and Clade III, the smallest, with 16 FLZs. Notably, the distribution trends for BcaFLZs, BniFLZs, and BolFLZs were similar, with a significant presence in Clade I, which included 17 BcaFLZs, 23 BniFLZs, and 17 BolFLZs. Each of the other three clades contained approximately equal numbers of the three *Brassica* FLZ types. Additionally, Clade II had the greatest number of OsFLZs, with 15 members, whereas Clade I contained 9 AtFLZ genes, more than the other subfamilies did. Notably, only one *AtFLZ* gene was placed into Clade III (Figure 1). All FLZs shared a common ancestor in each clade, suggesting that the diversity of plant FLZ proteins developed before the evolutionary split between monocots and dicots (Figure 1).

### 2.3. Analysis of Gene Structure and Identification of Conserved Motifs

To better elucidate the diversification of the *FLZ* genes in the three *Brassica* species, the exon/intron organization and conserved motifs of *FLZs* were analyzed. The exon/intron organization of the coding sequences of individual *FLZ* genes, including the number and length of exons/introns, is shown in Figure S1. The results indicate that all *FLZ* genes harbored only one intron except for *BcaFLZ14*, which consisted of five introns (Figure S1).

**Figure 2:**
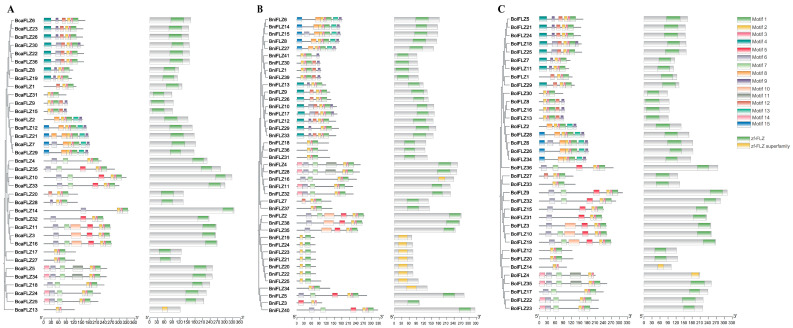
Conserved motif and domain analyses of the FLZs of the three *Brassica* species. (**A**) *Brassica rapa*; (**B**) *Brassica nigra*; and (**C**) *Brassica oleracea*. Fifteen conserved motifs of FLZ proteins were identified, and the size of each motif was proportional to the scale. The conserved domains of the FLZ proteins were examined via NCBI conserved domain search and SMART. The FCS-like zinc-finger domain (zf-FLZ) is represented by green boxes, and the zf-FLZ superfamily is represented by yellow boxes.

To further examine the structural diversity of *FLZ* in the three *Brassica* species, the conserved motifs of the 113 FLZ proteins were analyzed via MEME online software, and 15 different conserved motifs (motifs 1–15) were identified (Figure S2). The motif analysis revealed that nearly all of the FLZ proteins contained two motifs (motif 1 and motif 2), with the exception of BcaFLZ13, which contained only motif 2. Together with the results of the conserved structural domain analysis, we found that 101 FLZ proteins contained the zf-FLZ domain, whereas 12 FLZ proteins contained the zf-FLZ superfamily domain (Figure 2). The results of both analyses revealed that motifs 1 and 2 may represent the FCS-like zinc-finger domain. Furthermore, phylogenetic tree analysis revealed that genes within each subgroup presented unique gene structures and conserved motifs (Figure 2). Related genes in subgroups have similar motifs, indicating similar functions in the FLZ family.

Furthermore, recent research has shown that some FLZ proteins possess a conserved ATG8 (autophagy-related 8)-interacting motif (AIM) (EDYTLV) on the first β-sheet, which is highly conserved across plant species and plays a crucial role in autophagy and energy balance regulation [13]. Therefore, we used the FIMO-Motif search tool (https://meme-suite.org/meme/tools/fimo; accessed on 17 August 2024) to identify which FLZ proteins also contained AIM motifs in the three *Brassica* species [34]. Finally, we identified 13, 11, and 13 FLZ proteins containing AIM motifs in *Brassica rapa*, *Brassica nigra*, and *Brassica oleracea*, respectively (Table S2). The results indicated that the core AIM sequences and their surrounding residues were highly conserved across the three *Brassica* species (Figure S3).

### 2.4. Chromosome Distribution, Gene Duplication, and Collinearity Analysis of FLZ Genes

Gene localization helps in elucidating the function and role of genes and their interactions with each other. There are 10 chromosomes (2n = 2X = 20) in the *Brassica rapa* genome (Figure S4A), 8 chromosomes (2n = 2X = 16) in the *Brassica nigra* genome (Figure S4B), and 9 chromosomes (2n = 2X = 18) in the *Brassica oleracea* genome (Figure S4C). This study analyzed the genomic distribution of 113 *FLZs* across the three *Brassica* species via data from the *Brassica* genome database, which revealed that the *FLZ* genes are located on multiple chromosomes. Moreover, the *FLZ* genes were unevenly distributed across the chromosomes in *Brassica rapa* and *Brassica nigra*, including one gene, designated *BcaFLZ36*, located on contig00301 in *Brassica rapa* (Figure S4A,B). However, the 36 *FLZ* genes in *Brassica oleracea* were relatively evenly distributed across the nine chromosomes (Figure S4C, Table S3).

**Figure 3:**
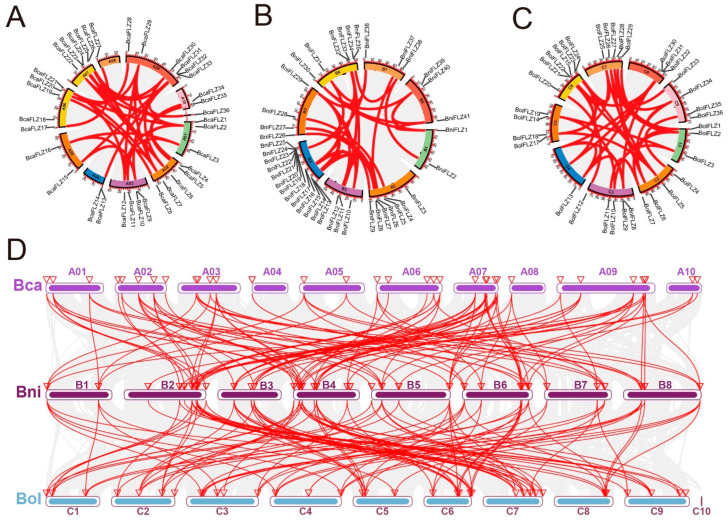
Gene position and collinearity analysis of the *FLZ* genes. Gene position and duplicated gene pairs in (**A**) *Brassica rapa*; (**B**) *Brassica nigra*; and (**C**) *Brassica oleracea*. Duplicated gene pairs are linked by red lines. (**D**) Synteny analyses of the *FLZ* genes between the three *Brassica* species. The gray lines indicate the synteny blocks between the genomes of the two species, whereas the red lines represent the syntenic pairs of the *FLZ* gene family between the genomes of the two species. A01–A10, B1–B8, and C1–C10 represent the chromosomes of the three species.

During evolution, chromosomes are amplified in different genomic regions through fragment duplication, tandem duplication, and genome-wide duplication (WGD), which help expand gene families [35]. In this study, we analyzed gene duplication within the *FLZ* gene family via TBtools II software (https://github.com/CJ-Chen/TBtools; accessed on 20 April 2024) [36]. As depicted in Figure 3A–C, a total of 109 gene pairs across the three *Brassica* species presented signs of duplication events, with these gene pairs located on different chromosomes. Specifically, 36, 36, and 37 duplication events were identified in *Brassica rapa*, *Brassica nigra*, and *Brassica oleracea*, respectively (Table S4). *Brassica* species have undergone WGT events during their evolution [26], suggesting that these pairs of *FLZ* genes likely originated from WGD. Moreover, high-density *FLZ* gene clusters have been identified in certain chromosomal regions, such as chromosome 4 of *Brassica nigra*, which includes seven *BniFLZ* genes, *BniFLZ19–BniFLZ25*, spanning positions 48,783 kb to 48,891 kb. A chromosomal segment of no more than 200 kb that includes two or more genes is classified as a tandem duplication [37], which suggests that these *FLZ* genes may have originated from tandem duplication. In addition, we observed five additional tandem duplication events involving ten *FLZ* genes in the three *Brassica* species: *BcaFLZ13* and *BcaFLZ14*; *BcaFLZ32* and *BcaFLZ33*; *BniFLZ34* and *BcaFLZ35*; *BolFLZ14* and *BolFLZ15*; and *BolFLZ31* and *BolFLZ32* (Figure S4). Previous studies have indicated that WGD and tandem duplication exhibit a compensatory relationship in gene expansion, wherein WGD simultaneously increases the dosage of all genes, whereas tandem duplication promotes the formation of gene clusters on chromosomes [29]. Following WGD, the redundancy introduced by duplication may diminish the necessity for additional gene expansion via tandem duplication, resulting in a reduction in tandem gene arrays [29], which is consistent with the findings presented in this study. Taken together, these results suggest that the expansion of the *FLZ* gene family in *Brassica* species mainly originated from WGD, followed by tandem duplication.

We subsequently calculated the Ka/Ks ratio to gain a deeper understanding of the selective pressures on the *FLZ* genes of the three *Brassica* species. Generally, a Ka/Ks ratio greater than 1 indicates adaptive evolution under positive selection, a ratio of 1 indicates neutral selection, and a ratio less than 1 indicates negative or purifying selection [38]. In our study, the Ka/Ks ratios for the duplicated gene pairs were all less than 1 (Table S4), suggesting that the *FLZ* gene family has undergone purifying selection and maintained a high degree of conservation throughout its evolutionary history. This finding is consistent with earlier results from Ka/Ks analyses of *FLZ* genes in various plant species, including *Arabidopsis*, rice, potato, eggplant, and pepper [7,39].

To further investigate the evolutionary relationships among the three *Brassica* species, the genomes of *Brassica rapa* (Bca) and *Brassica nigra* (Bni) were analyzed alongside the *Brassica oleracea* (Bol) genome to examine the collinearity relationships within the *FLZ* gene family. Among the three *Brassica* species, a total of 316 collinear gene pairs were identified (Figure 3D, Table S5), which included 108 pairs between *Brassica rapa* and *Brassica oleracea*, 105 pairs between *Brassica oleracea* and *Brassica nigra*, and 103 pairs between *Brassica rapa* and *Brassica nigra*. Additionally, we examined the genomic collinearity between *Brassica oleracea* and *Arabidopsis thaliana* and identified 40 pairs (Table S5).

### 2.5. Cis-Acting Regulatory Elements in the Promoters of BolFLZ Genes

Chinese kale, which is native to southern China, is a significant vegetable among the *Brassica oleracea* species and is classified as part of the CC genome type [33,40]. In this study, our detailed research focused on the *BolFLZ* genes of Chinese kale among the three *Brassica* species analyzed herein.

**Figure 4:**
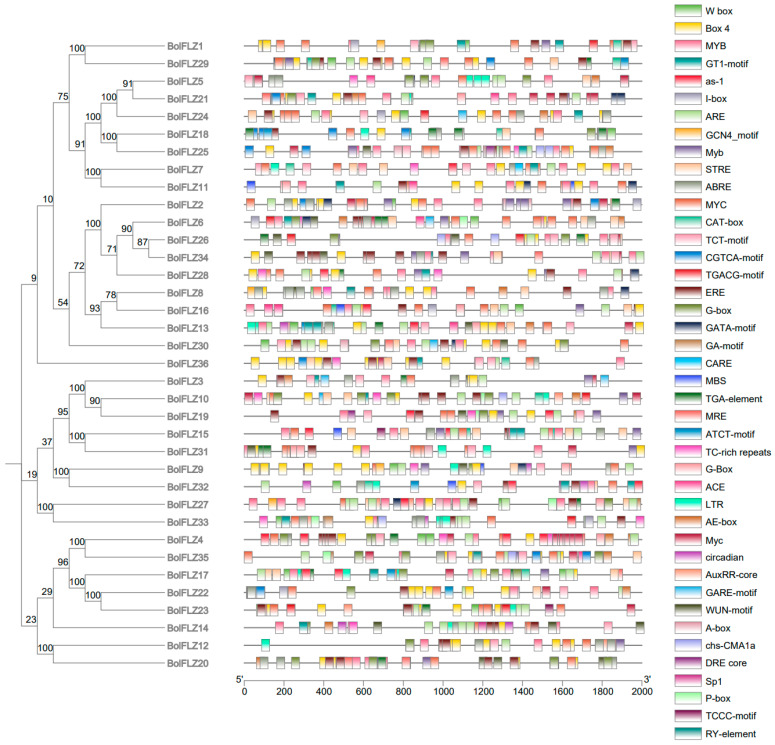
Analysis of cis-acting elements in the promoter regions of *BolFLZ.* The 2000 bp promoter region (upstream of the 5′UTR) of each *BolFLZ* gene was analyzed for cis-acting elements. Different cis-acting elements are indicated by different colors.

Gene expression in distinct biological processes is regulated by upstream transcription factors that bind to specific promoter-associated cis-acting elements. Cis-acting elements play pivotal roles in gene expression regulation, and their identification is crucial for understanding the direct or indirect regulatory influences of specific factors. This process will deepen our understanding of the complexities of gene expression divergence, overlap, and redundancy within the *FLZ* gene family. Therefore, to explore the biological function of the *BolFLZ* genes, the 2000 bp upstream sequences of 36 *BolFLZ* genes were extracted via TBtools II software (https://github.com/CJ-Chen/TBtools; accessed on 20 April 2024) and then submitted to the PlantCARE platform for cis-acting regulatory element prediction. As shown in Figure 4 and Table S6, 36 *BolFLZ* genes were found to contain a total of 42 different cis-acting elements, which included the following: (1) hormone response elements sensitive to ABA, methyl jasmonate (MeJA), gibberellic acid (GA), and auxin; (2) stress-related elements associated with conditions such as drought, cold, and anaerobic induction; (3) cis-acting elements involved in plant growth and development; and (4) cis-acting elements related to light responsiveness. Taken together, the diversity of these regulatory elements suggests that the *BolFLZ* gene family is closely associated with a wide range of biological activities and may act as key regulators, linking various hormone signaling pathways with other important biological functions.

### 2.6. Expression Patterns of the BolFLZ Genes in Various Chinese Kale Tissues

**Figure 5:**
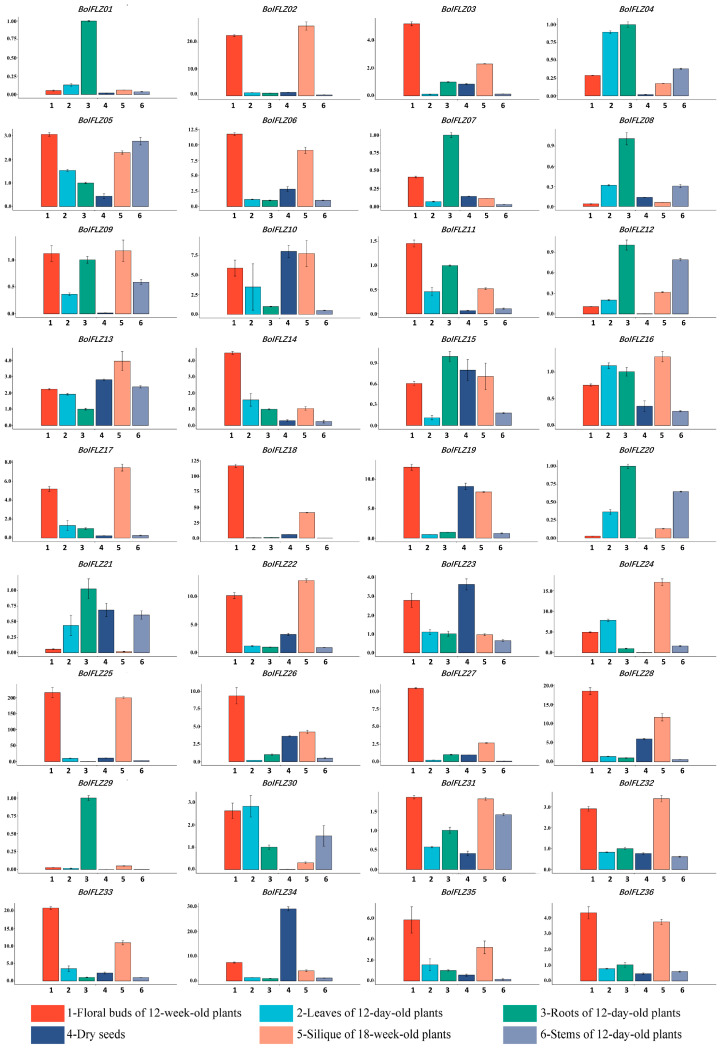
Expression profiles of the *BolFLZ* genes in different tissues. The expression profile data for the *BolFLZ* genes in various Chinese kale tissues were obtained through qRT–PCR. All analyses used *BolTubulin8* as an internal reference gene. The data shown are the means ± SDs of three biological replicates.

The spatial expression pattern of a gene provides critical insights into its possible functions and regulatory roles within an organism. To elucidate the tissue-specific expression patterns of the *BolFLZ* genes in Chinese kale, we conducted qRT–PCR analysis to assess their relative expression levels across various tissues. We analyzed the tissue-specific expression profiles of the *BolFLZ* genes in various organs of Chinese kale, including the roots, leaves, and stems of 12-day-old plants; the floral buds of 12-week-old plants; the siliques of 18-week-old plants; and dry seeds, providing a broad representation of the plant’s tissue types (Figure 5). Notably, most *BolFLZ* genes presented elevated expression levels in floral buds, roots, and siliques, whereas their expression was relatively low in leaves and dry seeds. Certain *BolFLZ* genes presented pronounced tissue-specific expression. For example, *BolFLZ01*, *BolFLZ08*, and *BolFLZ29* were specifically and highly expressed in the roots of 15-day-old seedlings, with expression levels ranging from 3~100-fold higher than those in other tissues. In contrast, *BolFLZ03*, *BolFLZ14*, *BolFLZ18*, *BolFLZ26*, and *BolFLZ27* were preferentially expressed in floral buds, where their expression levels were 2~110-fold greater than those in the other tissues. Furthermore, *BolFLZ34* exhibited high and specific expression in dry seeds, with levels 3~30-fold higher than those in other tissues (Figure 5). qRT–PCR analysis revealed these differential expression patterns, suggesting diverse roles for *BolFLZ* genes in tissue-specific developmental processes and physiological functions within Chinese kale.

### 2.7. Subcellular Localization of Six Typical BolFLZ Proteins

**Figure 6:**
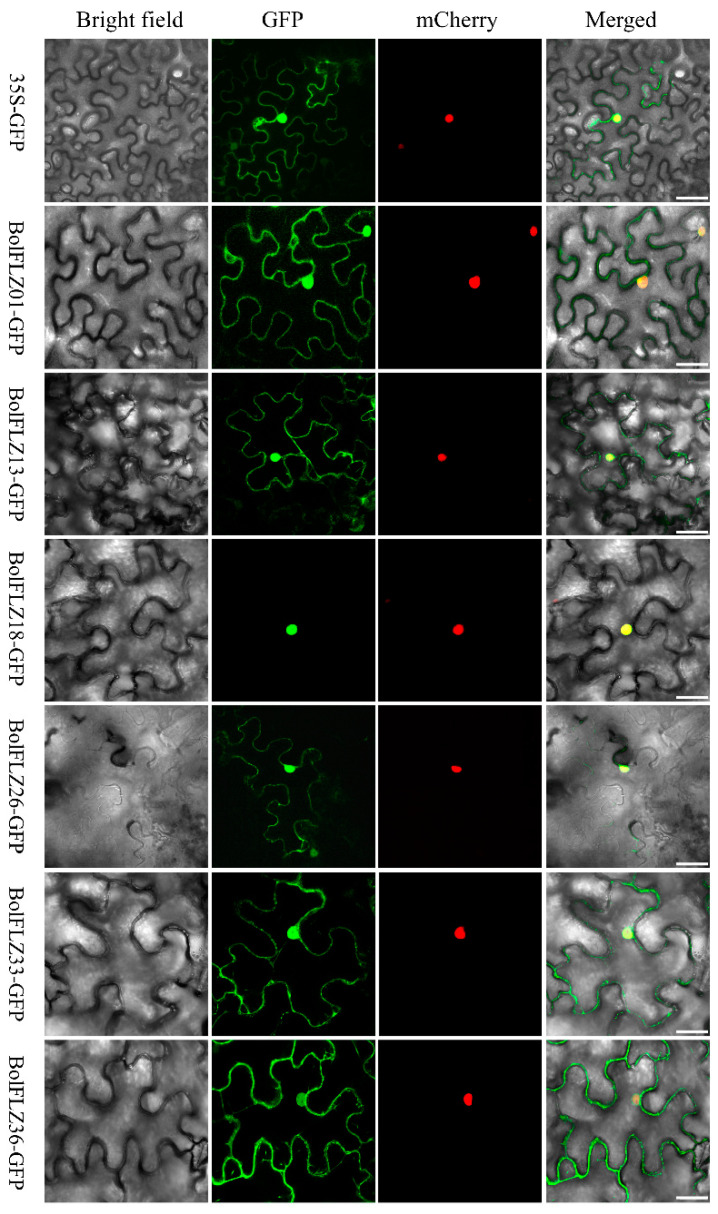
Subcellular localization of the six selected BolFLZ proteins. The BolFLZ-GFP fusion proteins, which were transiently expressed in tobacco (*Nicotiana benthamiana*) leaves, were observed via laser scanning confocal microscopy. The *35S-BolFLZ-GFP* constructs and *NLS-mCherry* (a nuclear marker) were cotransformed into the epidermal cells of tobacco. The *35S-GFP* construct served as a control. Scale bars represent 50 μm.

The subcellular localization of a protein is essential for comprehending its function and significantly contributes to functional gene studies. Bioinformatics analyses indicated that BolFLZ proteins may localize in various cellular compartments, including the nucleus, cytoplasm, chloroplasts, and mitochondria (Table S1). To validate this prediction, we selected six representative BolFLZ proteins across four distinct clades (Figure 1): three proteins from clade I, the largest, and one protein from each of the remaining clades. NLS-mCherry served as a control for the nuclear localization signal, and six-week-old *Nicotiana benthamiana* leaf epidermal cells were employed to ascertain the subcellular localization of these proteins. Additionally, 35S-GFP was separately transformed as an additional control to verify that the detected subcellular localization patterns were not due to artifacts from the transformation process or the GFP tag. Confocal laser scanning microscopy revealed that the green fluorescent protein (GFP) fusion protein signals for BolFLZ1, 13, 26, 33, and 36 were present in both the nucleus and cytoplasm, resembling the distribution pattern of GFP alone (Figure 6). Notably, the BolFLZ18-GFP signals were exclusively localized to the nucleus (Figure 6). These findings are generally consistent with prior bioinformatics predictions, indicating that these proteins predominantly operate within the nucleus and cytoplasm, in line with observations in other species [7,11].

### 2.8. Interactions of BolFLZ Proteins with BolSnRK1 Proteins

**Figure 7:**
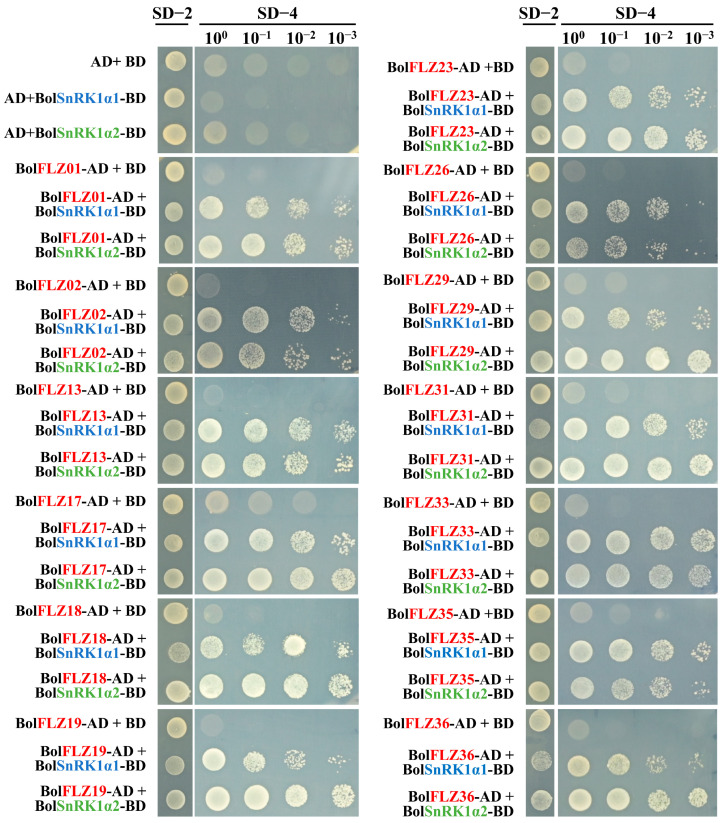
Thirteen selected BolFLZs interact with BolSnRK1α1 and BolSnRK1α2 in yeast cells. BolSnRK1α1 and BolSnRK1α2 were fused to the activation domain (AD), and the full-length BolFLZ protein was fused to the DNA-binding domain (BD). Cotransformed yeast clones were drip-inoculated onto SD/-Leu-Trp (SD-2) and SD/-Leu-Trp-His-Ade (SD-4) plates to test the interaction and subsequently cultured in a growth chamber.

Previous studies have shown that FLZ proteins can interact with the catalytic α subunits of SnRK1 in Arabidopsis, maize, and rice [5,15,16,22]. In this study, we performed a BLASTP search of the Chinese kale genome using the protein sequence of AtSnRK1.1 (AT3G01090.1) as a query and successfully identified two genes encoding the catalytic α subunit of SnRK1, hereafter referred to as BolSnRK1α1 and BolSnRK1α2. To explore the potential interactions between the BolFLZ proteins and the BolSnRK1α1 and BolSnRK1α2 proteins, a yeast two-hybrid assay was conducted. Thirteen BolFLZs from four distinct groups were chosen for this study. The selected proteins included BolFLZ01, BolFLZ17, BolFLZ18, BolFLZ23, BolFLZ29, BolFLZ33, and BolFLZ35 from Group I; BolFLZ02 and BolFLZ26 from Group II; BolFLZ13 from Group III; and BolFLZ19, BolFLZ31, and BolFLZ36 from Group IV (Figure 1). Furthermore, these proteins display a range of physicochemical properties and predicted subcellular localizations (Table S1), ensuring that the selected FLZ proteins accurately represent the broader FLZ gene family in Chinese kale. To this end, the full-length BolFLZs were fused to the GAL4 activation domain (AD), and BolSnRK1α1 and BolSnRK1α2 were fused to the DNA-binding domain (BD), resulting in the AD-BolFLZs and BD-BolSnRK1α1/α2 vectors, respectively. As anticipated, all thirteen AD-BolFLZs interacted with BD-BolSnRK1α1 and BD-BolSnRK1α2 but not with the empty BD vector (Figure 7). As shown in Figure 7, most of the BolFLZ proteins presented more robust growth in yeast cells cotransformed with BolSnRK1α2 than in those cotransformed with BolSnRK1α1 on high-strength selective media (SD–Trp/–Leu/–His/–Ade, SD-4), suggesting a potentially stronger binding affinity between the BolFLZ proteins and the BolSnRK1α2 protein. In contrast, BolFLZ26 displayed a weaker interaction with BolSnRK1α2 than with BolSnRK1α1, as indicated by the reduced growth of yeast transformants on SD-4 medium plates (Figure 7), highlighting the variability in protein–protein interaction strengths within the BolFLZ family.

### 2.9. Expression Patterns of the BolFLZs in Response to Abiotic Stresses

Chinese kale is classified as an environmentally sensitive leafy vegetable and is particularly susceptible to abiotic stresses [40,41]. In the Arabidopsis genome, a total of 19 *FLZ* genes have been identified, the majority of which exhibit active responses to various stress treatments [17]. Previous studies have reported that *FLZ* genes in other plant species are also closely associated with stress responses [5,11,22]. According to cis-acting element analysis, all the *BolFLZ* promoters contained cis-acting elements associated with stress responsiveness (Figure 4). To elucidate the potential functions of the *BolFLZ* gene family, we analyzed the expression patterns of 15 selected *BolFLZ* genes from four distinct subfamilies under different abiotic stresses, including heat, cold, drought, and salt, via qRT–PCR. Given that ABA is the most critical stress phytohormone [42], we also examined the responsiveness of the *BolFLZ* genes to ABA treatment.

**Figure 8:**
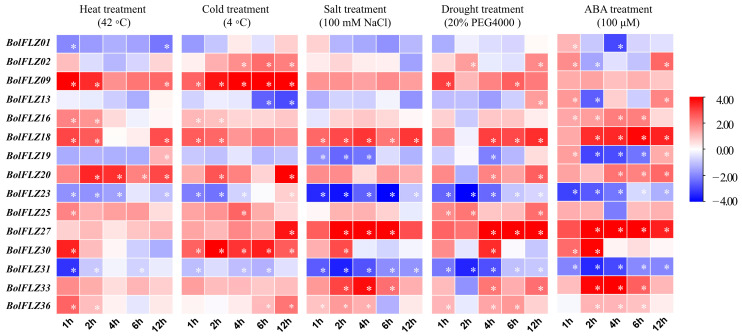
Analysis of *BolFLZ* gene expression under abiotic stress conditions via qRT–PCR. Heat (42 °C), cold (4 °C), salt (NaCl), drought (PEG4000), and hormone (ABA) stresses were applied to 12-day-old Chinese kale plants to analyze the responsiveness of the *BolFLZ* genes. Samples collected from well-watered Chinese kale plants served as controls. The relative expression levels of the *BolFLZ* genes were determined via qRT–PCR following treatment durations of 1 h, 2 h, 4 h, 6 h, and 12 h. The expression levels of related genes were calculated via the 2^−ΔΔCt^ method, with 0 h used as a control. The data were subsequently converted to log2FC values and visualized via TBtools II. The values for each time point represent the means of three biological replicates. The red and blue colors indicate increased and decreased expression levels, respectively, relative to those in the control. * refers to significant differences with *p* < 0.05 compared with the corresponding 0 h controls.

The results indicated that the expression levels of the *BolFLZ* genes could be influenced by various stress treatments, and these genes appeared to respond relatively consistently to different stress treatments, with 10 genes upregulated and 5 genes downregulated (Figure 8). Under heat treatment, the expression levels of 6 genes—*BolFLZ09*, *BolFLZ18*, *BolFLZ20*, *BolFLZ25*, *BolFLZ27*, and *BolFLZ33*—increased 3- to 20-fold, whereas the expression levels of *BolFLZ01*, *BolFLZ23*, and *BolFLZ31* significantly decreased. In contrast, the expression of *BolFLZ19* initially decreased before increasing, whereas the expression of *BolFLZ30* initially increased before decreasing. Cold stress also had a strong effect on the expression of these 15 *BolFLZ* genes, with most being significantly upregulated, including *BolFLZ02*, *BolFLZ09*, *BolFLZ16*, *BolFLZ18*, *BolFLZ20*, *BolFLZ25*, *BolFLZ27*, *BolFLZ33*, and *BolFLZ36*, among which *BolFLZ09* and *BolFLZ30* presented the most significant increases. In contrast, the expression levels of *BolFLZ13* and *BolFLZ31* significantly decreased. Additionally, the expression levels of *BolFLZ01* and *BolFLZ23* were downregulated during the initial phase of cold treatment and subsequently upregulated after 12 h. Under salt treatment, the expression levels of *BolFLZ09*, *BolFLZ18*, *BolFLZ20*, *BolFLZ27*, and *BolFLZ33* were elevated at nearly all the analyzed time points. The expression levels of *BolFLZ19*, *BolFLZ23*, and *BolFLZ31* significantly decreased following salt treatment. PEG-simulated drought stress (20% PEG 4000) resulted in increased expression levels of *BolFLZ09*, *BolFLZ18*, *BolFLZ25*, and *BolFLZ27* at most analyzed time points. The expression levels of *BolFLZ01*, *BolFLZ23*, and *BolFLZ31* were inhibited by PEG. Interestingly, the expression levels of *BolFLZ20*, *BolFLZ33*, and *BolFLZ36* decreased after 2 h of PEG4000 treatment but subsequently increased at the other analyzed time points.

Finally, we assessed the response of these 15 *BolFLZ* genes to ABA. The results demonstrated that the expression levels of *BolFLZ16*, *BolFLZ18*, *BolFLZ20*, *BolFLZ27*, *BolFLZ33*, and *BolFLZ36* were significantly upregulated following ABA treatment, whereas the expression levels of *BolFLZ23* and *BolFLZ31* were significantly inhibited by ABA treatment. The expression level of *BolFLZ01* increased during the first hour of ABA treatment but subsequently decreased. The expression levels of *BolFLZ02*, *BolFLZ13*, *BolFLZ19*, and *BolFLZ25* decreased at specific time points but increased at 1 h and 12 h after ABA treatment. These results suggest that different *BolFLZ* genes may play distinct roles in the stress response to various environmental stresses.

As described above, most *BolFLZ* genes tended to be upregulated in response to all abiotic stresses in this study (Figure 8), suggesting that the *BolFLZ* gene family may play a positive role in stress tolerance in Chinese kale. Notably, five members (*BolFLZ09*, *BolFLZ18*, *BolFLZ20*, *BolFLZ27*, and *BolFLZ30*) were significantly upregulated in response to all the treatments. Among them, *BolFLZ30* stands out for its robust induction, ranging from 10- to 40-fold greater than that of the control samples under the various stress treatments, suggesting a significant role in mediating stress responses in Chinese kale. In contrast, the expression levels of two members (*BolFLZ23* and *BolFLZ31*) were significantly inhibited under various stress conditions, suggesting that these genes may be negatively regulated in such scenarios. However, only a few genes, such as *BolFLZ02*, exhibit disordered expression patterns under abiotic stress.

Furthermore, the expression levels of the *BolFLZ* genes appear to be more sensitive to ABA treatment than to other stress treatments (Figure 8). This heightened sensitivity suggests that the *BolFLZ* genes may play a pivotal role in the ABA signaling pathway in plants, which is essential for adapting to environmental stresses such as drought, cold, and high salinity. The ABA-induced changes in the expression of the *BolFLZ* genes may represent a key mechanism through which plants fine-tune their stress responses, potentially leading to more efficient adaptation and survival strategies under adverse conditions. However, further research is necessary to validate these findings and explore this hypothesis in future studies.

Taken together, the varied expression patterns of *BolFLZ* genes under different abiotic stresses suggest the complex involvement of this gene family in stress responses, highlighting the need for further investigation into their role in plant abiotic stress.

## 3. Discussion

### 3.1. Conservation and Evolution of the FLZ Gene Family in Brassica Species

*The FLZ* genes constitute a poorly studied gene family that is widely present in plants, and their biological functions are only just beginning to be explored [2,13]. Molecular function studies on the *FLZ* genes of Arabidopsis and rice have revealed the crucial role of this gene family in regulating plant growth and development as well as stress responses [8,9,13,22]; however, this gene family has not been examined in *Brassica* species, which are classical model organisms for investigating polyploid evolution [30]. These species have gained increasing attention because of their significant economic value. Previous studies have shown that Arabidopsis and the *Brassica* lineage diverged ~24 million years ago [25]. *Brassica* subsequently underwent a unique WGT, followed by extensive gene loss, gene acquisition, and chromosomal rearrangements involving partial duplications and deletions [26]. Although complete genome sequences are available, the *FLZ* genes in *Brassica* species have yet to be identified and comprehensively analyzed. To further our understanding of the *FLZ* gene family, we systematically analyzed *FLZ* family genes in three diploid *Brassica* species (*Brassica rapa*, *Brassica nigra*, and *Brassica oleracea*) and discovered potential candidate genes for further functional analysis.

The number of *FLZ* genes varies significantly among species, with 19 identified in Arabidopsis [1], 37 in maize [5], 29 in rice [22], 19 in tomato [7], and 21 in liquorice [11]. In this study, a total of 113 *FLZ* genes were identified across the three *Brassica* species, including 36 each in *Brassica rapa* and *Brassica oleracea* and 41 in *Brassica nigra* (Figure 1 and Table S2). These *Brassica* species have a similar number of *FLZ* genes as maize but significantly more than most other plants. The variations in the number of *FLZ* genes among species may arise from the evolutionary and domestication processes of these species. Furthermore, structural characterization revealed that these *FLZ* genes had highly conserved structural domains; however, the protein length, number of motifs, and composition of each family varied significantly (Figure 2). Notably, excluding *BcaFLZ14*, which contains five introns, the 112 *FLZ* genes possess only one intron (Figure S1), indicating that *FLZ*s are associated with the response to both biotic and abiotic stress since genes responsive to stress exhibit fewer introns [43]. In this study, the FLZ proteins in the *Brassica* species were grouped into four monophyletic branches on the basis of protein sequence identity (Figure 1), which is consistent with previous findings in four *Solanaceae* species [7]. In addition, different subgroups may be responsible for varying functions.

Gene duplication not only expands the genome content but also diversifies gene function to ensure the optimal adaptability and evolution of plants [35,44]. In this study, 109 gene pairs were found to be collinear and dispersed across different chromosomes (Figure 4), suggesting that WGD significantly contributed to the expansion of the *FLZ* family in the three *Brassica* species. Moreover, the greater number of *FLZ* paralogues in *Brassica* is consistent with the extensive history of gene/genome duplication events in this species [26]. Theoretically, the number of *FLZ* genes in *Brassica* species is expected to be three- to six-fold greater than that in Arabidopsis because of WGT events. However, the reduced copy number of FLZ proteins in the three diploid *Brassica* species suggests that certain members of the *FLZ* family may have been lost or undergone genetic alterations during evolution, as previously mentioned [45,46]. This loss or alteration may be due to the fact that, during the prolonged course of natural selection, the number of *FLZ* genes was likely sufficient for *Brassica*, resulting in the nonretention of some duplicated *FLZ* genes within the *Brassica* genome. The duplicates retained in the genome primarily provided adaptive advantages, including enhanced stress tolerance and reproductive benefits under challenging conditions [28].

### 3.2. FLZ Proteins Mediate Protein Interactions

The FLZ domain is regarded as the canonical protein–protein interaction module of FLZ proteins, enabling these proteins to serve as molecular scaffolds that promote extensive interactions with diverse proteins, such as kinases and transcription factors, thereby contributing significantly to numerous cellular processes [39,47]. Among these proteins, SnRK1 is recognized as the first protein identified to interact with FLZ domain-containing proteins, resulting in the formation of a complex that plays a critical role in regulating glucose homeostasis, developmental transitions, and stress signaling through its interaction with the SnRK1α subunit [1,5,15]. For example, OsFLZ18 specifically binds to SnRK1A, thereby suppressing the expression of the αAmy3 gene, a crucial positive regulator of rice flooding tolerance [22]. In Arabidopsis, FLZ6 and FLZ10 form a complex with SnRK1 near the endoplasmic reticulum (ER) while modulating the levels of SnRK1α in response to energy availability [16]. In this study, we identified BolSnRK1α1 and BolSnRK1α2, two genes that encode the SnRK1 catalytic α subunits in Chinese kale, and demonstrated their significant interaction with BolFLZs through yeast two-hybrid analysis (Figure 8), which is consistent with previous reports in maize, tomato, and liquorice [5,7,11]. However, the underlying molecular mechanism remains elusive in these plants. Overall, these findings indicate that the SnRK1α-FLZ regulatory module is conserved in Chinese kale, highlighting its potential as a fundamental component in the plant stress response and developmental mechanisms.

Recently, Yang et al. (2023) identified seven FLZ proteins in Arabidopsis as previously uncharacterized ATG8-interacting partners that function as repressors of SnRK1 signaling, negatively modulating autophagy and plant tolerance to carbon starvation. In addition to those in Arabidopsis, the ATG8-FLZ-SnRK1 regulatory modules in maize have been confirmed to regulate plant tolerance to energy starvation and appear to be highly conserved in seed plants [13]. Protein sequence analysis revealed that the canonical EDYTLV sequence within the FLZ domain, referred to as the AIM, is crucial for determining whether FLZ binds to ATG8 [13]. In this study, we identified 37 FLZ proteins containing AIM motifs (EDYTLV) in the three *Brassica* species (Table S2) that exhibit a highly conserved AIM (Figure S3). Considering the conservation of the ATG8-FLZ-SnRK1 regulatory axis throughout the evolutionary history of seed plants, we suspect that AIM-containing FLZs might interact with ATG8 in *Brassica* species. However, this hypothesis requires future experimental validation.

Furthermore, research indicates that the FLZ proteins exhibit homo- and heterodimerization properties. In Arabidopsis, a yeast two-hybrid assay demonstrated that FLZ7, FLZ10, FLZ12, and FLZ15 exhibit homologous dimerization and strong heterodimerization with other FLZ proteins, whereas FLZ1, FLZ6, and FLZ11 interact specifically with only one or two FLZ proteins [15]. These multiprotein complexes may possess promiscuous interaction properties that facilitate the recruitment of additional subunits to the complex [15]. In plants, including *Brassica* species, we hypothesize that there is a high probability of selective interactions among FLZ proteins, potentially leading to the assembly of homo- and heterodimers. However, to our knowledge, similar reports have not been published in species besides Arabidopsis.

### 3.3. Potential Roles of BolFLZ Family Genes in Abiotic Stress Responses

Chinese kale (*Brassica oleracea* var. *alboglabra*), which is native to China and cultivated primarily in South China and Southeast Asia, is valued for its flavor and nutritional benefits, including its ability to produce vitamin C, carotenoids, phenolics, and glucosinolates [33,48]. However, similar to other *Brassica* vegetables, Chinese kale is highly sensitive to abiotic stress, such as extreme temperatures, drought, and altered salinity, which frequently results in reduced yield and quality [40]. Hence, identifying genes that respond to abiotic stress is a critical initial step towards breeding cultivars with improved stress tolerance.

The available evidence indicates a significant role for the *FLZ* gene family in plant stress tolerance and adaptive growth [17]. For example, TaFLZ2D functions as a positive regulator of salt stress tolerance by enhancing ion stress tolerance and reactive oxygen species (ROS) detoxification capabilities in wheat [6]. OsFLZ18 negatively regulates flood tolerance in rice by interacting with SnRK1A and suppressing its transcriptional activation of the αAmy3 gene [22]. Virus-induced gene silencing (VIGS) experiments indicated that both SlFLZ2 and SlFLZ18 play positive roles in heat tolerance in tomato [7]. Despite these findings, the precise roles of the FLZ proteins in the growth and development of *Brassica* species, particularly in response to abiotic stress, remain to be fully characterized.

Bioinformatic analysis revealed that the promoters of the *BolFLZ* genes contain various cis-regulatory elements involved in regulating plant growth and development, hormone signal transduction, and stress responses. This finding is supported by qRT–PCR evidence that the expression of the *BolFLZ* genes fluctuates significantly under various abiotic stresses (Figure 8). The majority of these genes are positively regulated by stress, whereas a minority, such as *BolFLZ23* and *BolFLZ31*, exhibit downregulated expression. However, how these genes are involved in regulating stress responses in Chinese kale requires further research and deserves further study in our future work, particularly the role of the FLZ proteins in heat tolerance, which is crucial because the sensitivity of Chinese kale to heat limits its distribution and production [40].

## 4. Materials and Methods

### 4.1. Plant Materials and Growth Conditions

Chinese kale (*Brassica oleracea* L. var. *alboglabra*) for tissue-specific expression analysis was cultivated in a glasshouse at 25 °C under a 16 h/8 h light/dark photoperiod. For the abiotic stress treatments, the seeds were surface sterilized in 75% (*v*/*v*) ethanol for 15 s, followed by 0.6% (*v*/*v*) sodium hypochlorite solution for 15 min, and then rinsed three times with sterile water. These sterilized seeds were cultured on 1/2 MS media for 7 days at 25 °C and then transferred to a simple hydroponic culture system utilizing 1/2 Hoagland nutrient solution (pH 5.8–6.0). After 5 days of growth, the seedlings were subjected to various treatments, including heat stress (42 °C), cold stress (4 °C), 100 mM NaCl (at 25 °C), 100 μM ABA (at 25 °C), and 20% PEG4000 (at 25 °C). Following treatments lasting 0 h, 1 h, 2 h, 4 h, 6 h, and 12 h, the leaves of three seedlings were pooled to form one sample, harvested, and frozen in liquid nitrogen for RNA isolation. All the experiments were conducted with three independent biological replicates.

### 4.2. Database Searches and Sequence Retrieval

The genomic data for Chinese cabbage (*Brassica rapa*; Bca), black mustard (*Brassica nigra*; Bni), and Chinese kale (*Brassica oleracea*; Bol) are sourced from the following links: http://tbir.njau.edu.cn/NhCCDbHubs/downloadFile_fileShow.action (accessed on 23 March 2024) [49], https://cruciferseq.ca/Bnigra_download (accessed on 23 March 2024) [27], and https://ngdc.cncb.ac.cn/gwh/Assembly/83516/show (accessed on 23 March 2024) [50]. *AtFLZ* and *AtSnRK1α* data were downloaded from the TAIR database (https://www.arabidopsis.org/; accessed on 20 March 2024). *OsFLZ* data were downloaded from the RGAP database (http://rice.plantbiology.msu.edu/; accessed on 20 March 2024).

To identify *FLZ* gene family members in the three *Brassica* species, we employed a multistep approach. Initially, we conducted a BLASTP search using previously reported Arabidopsis *FLZ* gene family protein sequences. Concurrently, we utilized the hidden Markov model (HMM) of the FLZ family protein structure obtained from the Pfam database (http://pfam.sanger.ac.uk; accessed on 28 March 2024) to search for protein sequences containing the characteristic domain (PF04570). Candidate genes were selected by intersecting the results from these two methods. We subsequently verified the structural domains of the candidate proteins via online analysis tools from Pfam (http://pfam.xfam.org/; accessed on 6 April 2024), NCBI Batch CD Search (https://www.ncbi.nlm.nih.gov/Structure/bwrpsb/bwrpsb.cgi; accessed on 6 April 2024), and SMART (http://smart.embl-heidelberg.de/; accessed on 6 April 2024). Finally, we manually screened nonredundant putative FLZ sequences, removing those lacking the complete FLZ domain or containing incomplete FLZ domains.

The physicochemical properties of the deduced FLZ protein sequences, including pI, MW, GRAVY, and hydrophilicity, were estimated via the ProtParam tool (https://web.ExPASy.org/protparam/; accessed on 10 April 2024). The subcellular localization of FLZ proteins in the three *Brassica* species was predicted via the WoLF PSORT web server (https://wolfpsort.hgc.jp/; accessed on 10 April 2024).

### 4.3. Sequence Alignment and Phylogenetic Analysis of the FLZ Proteins

The full-length sequences of FLZ proteins from three *Brassica* species, Chinese cabbage, black mustard, and Chinese kale, as well as Arabidopsis and rice, were used to construct phylogenetic trees via the MUSCLE method in the software MEGA X (version 11.0). Multiple sequence alignments were performed via the default parameters of ClustalW within the MEGA X program. The phylogenetic trees were constructed via the neighbor–joining (NJ) method on the basis of the p-distance model. Bootstrap analysis with 1000 replications was performed to validate the trees. The trees were then visualized via the online tool iTOL (https://itol.embl.de/upload.cgi; accessed on 15 November 2024).

### 4.4. Genomic Structure, Motif, and Domain Analyses of FLZ Members in the Three Brassica Species

To illustrate the exon/intron profiles of *FLZ* genes in the three *Brassica* species, the Gene Structure Display Server program (GSDS, http://gsds.gao-lab.org/; accessed on 20 April 2024) was used on the basis of a comparison between CDS and genomic DNA sequences. To better illustrate the structural relationships of the *FLZ* genes in the three *Brassica* species, the gene structures of the three *Brassica* species were visualized via TBtools II software (https://github.com/CJ-Chen/TBtools; accessed on 20 April 2024) [36].

For the identification of conserved motifs, the FLZ protein sequences from the three *Brassica* species were uploaded to the MEME program (http://meme-suite.org/tools/meme; accessed on 21 April 2024), setting the number of motifs to identify as 15, with other parameters remaining at their default values [51]. The results were visualized via TBtools II software (https://github.com/CJ-Chen/TBtools; accessed on 20 April 2024).

### 4.5. Analysis of the Cis-Regulatory Elements in the Promoters of FLZ Genes

TBtools II software (https://github.com/CJ-Chen/TBtools; accessed on 20 April 2024) was used to extract the promoter regions 2000 bp upstream of the translation start codons (ATG) in the three *Brassica* species [36]. The sequences were submitted to the online database PlantCARE (http://bioinformatics.psb.ugent.be/webtools/plantcare/html/; accessed on 22 April 2024) for analysis of cis-acting elements [52]. Gene information and cis-acting element information were submitted to the GSDS (http://gsds.gao-lab.org/; accessed on 22 April 2024) website to obtain a map of the cis-acting elements for visualization [53].

### 4.6. Subcellular Localization Assays in Nicotiana benthamiana Leaves

We assessed the subcellular localization of the BolFLZs through transient expression assays in the leaf epidermal cells of 6-week-old *Nicotiana benthamiana*. The full-length coding sequences (CDSs) of the *BolFLZs* were subsequently cloned and inserted into the binary vector pCambia1300-UBQ-GFP in frame with a GFP tag at the C-terminus; the primers used are listed in Table S7. The fusion constructs were cotransfected into tobacco leaves along with a nuclear localization marker (NLS-mCherry) via an agroinoculation assay. A Zeiss LSM 800 confocal laser scanning microscope (Germany) was used to determine the localization of the target proteins three days postinoculation.

### 4.7. RNA Isolation and Quantitative Real-Time PCR Analysis

Total RNA was extracted from multiple tissues of *Brassica* plants at different developmental stages via the Eastep™ Universal RNA Extraction Kit (Promega, Shanghai, China). Samples were collected from the roots, leaves, and stems of 12-day-old plants; the floral buds of 12-week-old plants; the siliques of 18-week-old plants; and dry seeds. The quality and concentration of the extracted RNA were assessed via a NanoDrop 2000 spectrophotometer (Thermo Fisher Scientific, Waltham, MA, USA). cDNA synthesis was performed using 2 μg of total RNA from each sample via the TransScript All-in-One First-Strand cDNA Synthesis Kit (Vazyme, Nanjing, China) following the manufacturer’s protocol. The synthesized cDNA was diluted and used as a template for quantitative real-time PCR (qRT–PCR). Specific primers for qRT–PCR were designed via the PrimerQuest Tool (https://sg.idtdna.com/PrimerQuest/Home/Index; accessed on 28 April 2024) and subsequently validated for specificity via TBtools II software (https://github.com/CJ-Chen/TBtools; accessed on 20 April 2024) (Table S7). The *BolTubulin8* housekeeping gene served as an internal control to normalize expression data and account for variations in sample loading and amplification efficiency [41]. Ct values for *BolTubulin8* ranging from 20 to 25 are considered effective. Reactions were conducted in a 10 μL volume containing diluted cDNA, specific primers, and SYBR Green PCR Master Mix (Vazyme, Nanjing, China). Amplification and detection were performed on a CFX96 real-time system (Bio-Rad, California, USA). The thermal cycling conditions were as follows: initial denaturation at 95 °C for 1 min, followed by 45 cycles of denaturation at 95 °C for 10 s, and annealing/extension at 60 °C for 15 s. The melting curve program consisted of a continuous increase in temperature from 60 °C to 95 °C, with continuous fluorescence measurements. The relative expression levels of the target genes were calculated via the comparative CT (2^−∆∆CT^) method. The specific primers used for each gene are detailed in Table S7.

### 4.8. Yeast Two-Hybrid Assay

The yeast two-hybrid assay was conducted according to the manufacturer’s protocols (Clontech, Mountain View, CA, USA). The cDNAs encoding the BolSnRK1α1 and BolSnRK1α2 proteins were subsequently cloned and inserted into the pGBKT7 vector to serve as bait constructs, and the cDNAs of the BolFLZ family members were subsequently cloned and inserted into the pGADT7 vector to serve as prey constructs. The specific primers used in these cloning procedures are detailed in Table S7. The yeast strain AH109 was cotransformed with different combinations of these vectors. Protein–protein interactions were assessed by cultivating the yeast transformants on synthetic dropout (SD/-His/-Trp) and synthetic quadruple dropout (SD/-His/-Trp/-Leu/-Ade) solid media and incubating them at 30 °C for 3–5 days to assess the growth of colonies. To assess the strength of these interactions, a serial dilution of the yeast suspension was conducted to establish a 10-fold concentration gradient, and yeast growth was monitored across varying dilution levels. Standardized photography was employed to maintain consistency in evaluating colony growth. All experiments were conducted with three biological replicates to ensure the reproducibility of the interaction strengths observed.

### 4.9. Statistical Analysis

Microsoft Excel 2021 was used for data entry and statistical analysis, and GraphPad Prism 9.5 (GraphPad Software, LLC, San Diego, CA, USA) was used to generate the bar graphs.

## 5. Conclusions

In this study, a total of 113 *FLZ* genes were identified in the three *Brassica* species and classified into four groups. Chromosomal mapping and synteny analysis revealed that the 113 *FLZ* genes were distributed across all chromosomes, with some gene clustering. WGD and tandem duplication were identified as the main causes of FLZ gene expansion in the three *Brassica* species. The *FLZ* genes all contain the zf-FLZ domain and have few introns, suggesting that they function as stress response factors. A total of 13 representative BolFLZ proteins from distinct groups interact with BolSnRK1α1 and BolSnRK1α2. Furthermore, qRT–PCR analysis revealed that the expression levels of the *BolFLZ* genes varied across different tissues and were significantly altered in response to heat, cold, drought, salt, or ABA stresses, highlighting their potential roles in environmental adaptation in Chinese kale. This study enhances our understanding of the *FLZ* genes in *Brassica* species and paves the way for further exploration of the functions of the *BolFLZ* genes in Chinese kale. Future studies should focus on elucidating the underlying mechanisms to investigate their potential roles in stress signaling pathways. Furthermore, investigating the interactions between the BolFLZ proteins and other key regulatory proteins may reveal novel mechanisms of stress response and adaptation in *Brassica* species.

## Data Availability

Data will be made available upon request.

## Supplementary Materials

The following supporting information can be downloaded at: https://www.mdpi.com/article/10.3390/ijms252312907/s1.
