# Supplementary material for: Genome-Wide Identification of FCS-Like Zinc Finger (FLZ) Family Genes in Three Brassica Plant Species and Functional Characterization of BolFLZs in Chinese Kale Under Abiotic Stresses
Source: Int J Mol Sci. 2024 Nov 30;25(23):12907. doi: 10.3390/ijms252312907 (PMC11641214; doi:10.3390/ijms252312907)
Supplement: Supplementary file 1 [file ijms-25-12907-s001.zip › ijms-3319722-supplementary/Figures S1-S4.pdf]

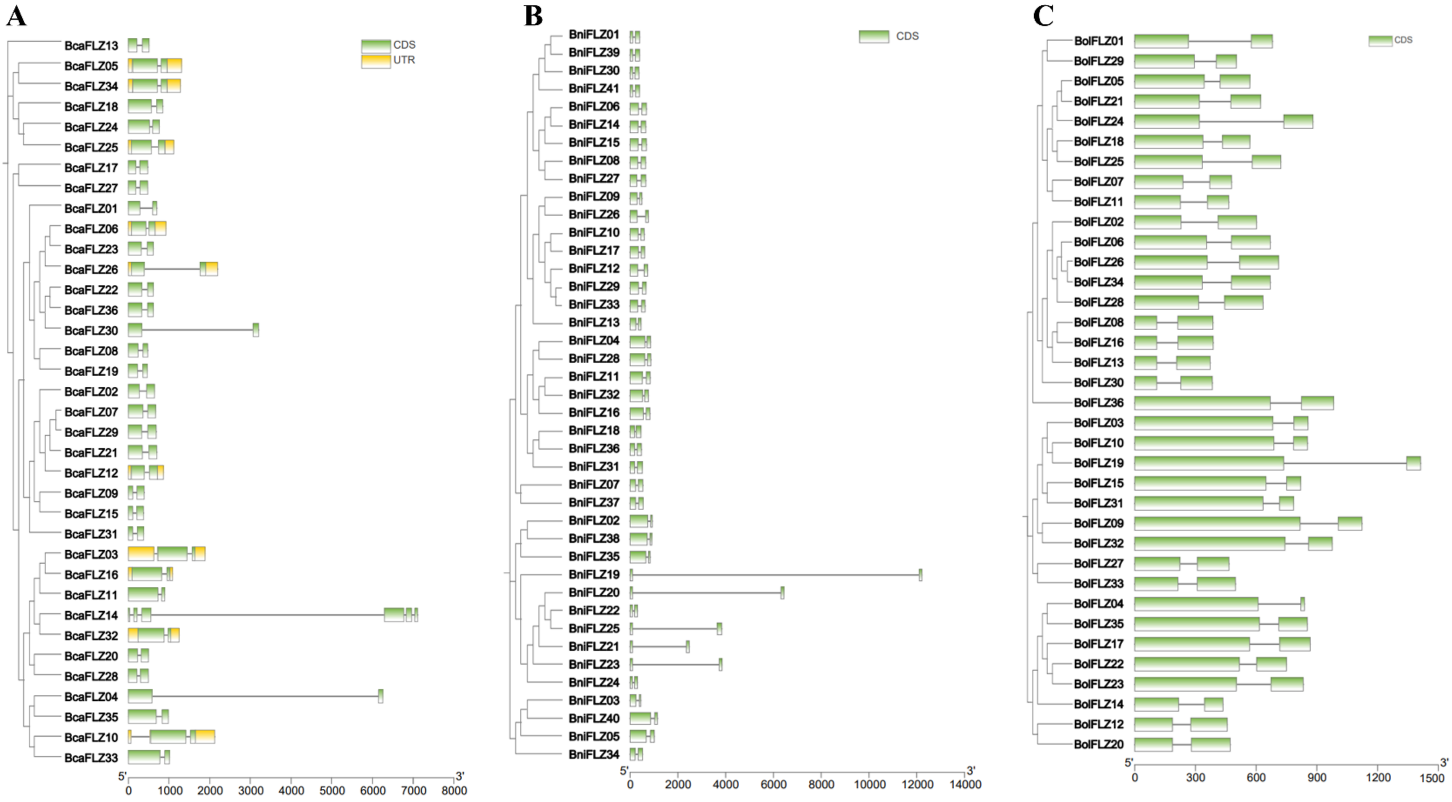

Figure S1 Exon–intron organization of the FLZ genes of three *Brassica* species. (A) Chinese cabbage (*Brassica rapa*; Bca); (B).black mustard (*Brassica nigra*; Bni); (C) Chinese kale (*Brassica oleracea*; Bol). Green boxes indicate exons, yellow boxes indicate untranslated regions (UTRs), and gray lines indicates introns.

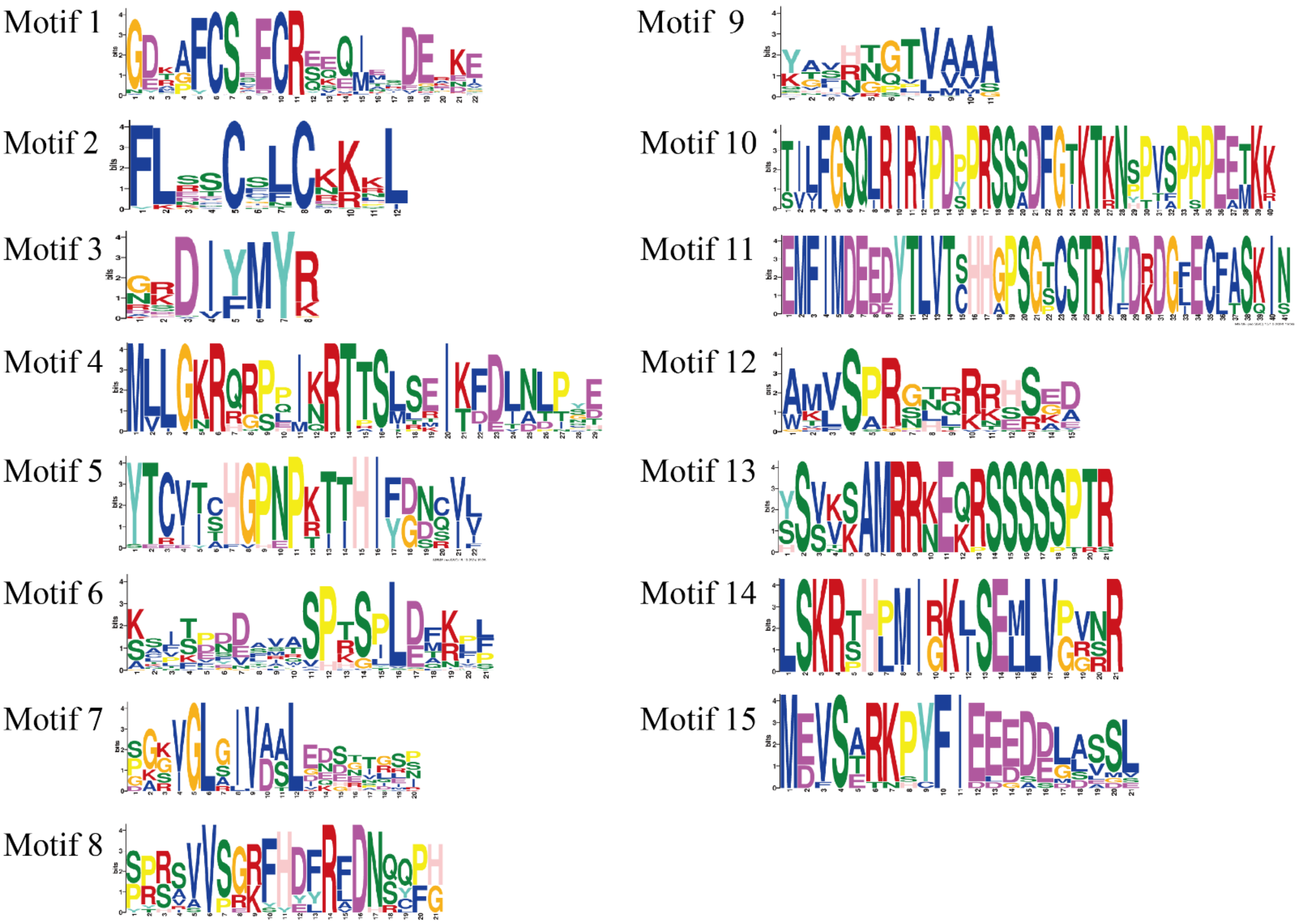

Figure S2 The fifteen conserved motif structures in three *Brassica* species. The conserved motifs were determined by MEME suite (http://meme-suite.org/tools/meme)

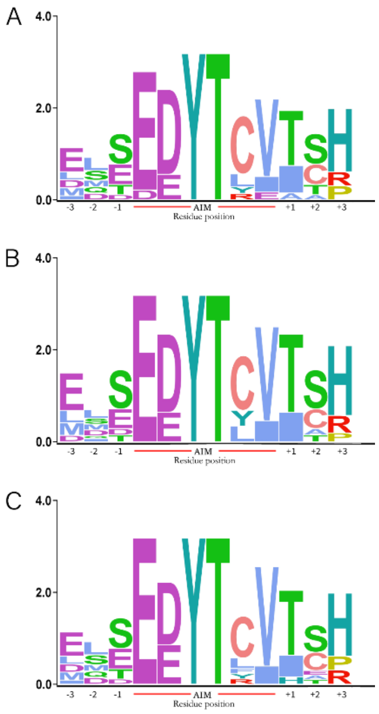

Figure S3 The FLZ-AIM motif structures in three *Brassica* species. (A) Chinese cabbage (*Brassica rapa*; Bca); (B) Black mustard (*Brassica nigra*; Bni); (C) Chinese kale (*Brassica oleracea*; Bol). The consensus FLZ-AIM sequence identified by FIMO-Motif (https://meme-suite.org/meme/tools/fimo).

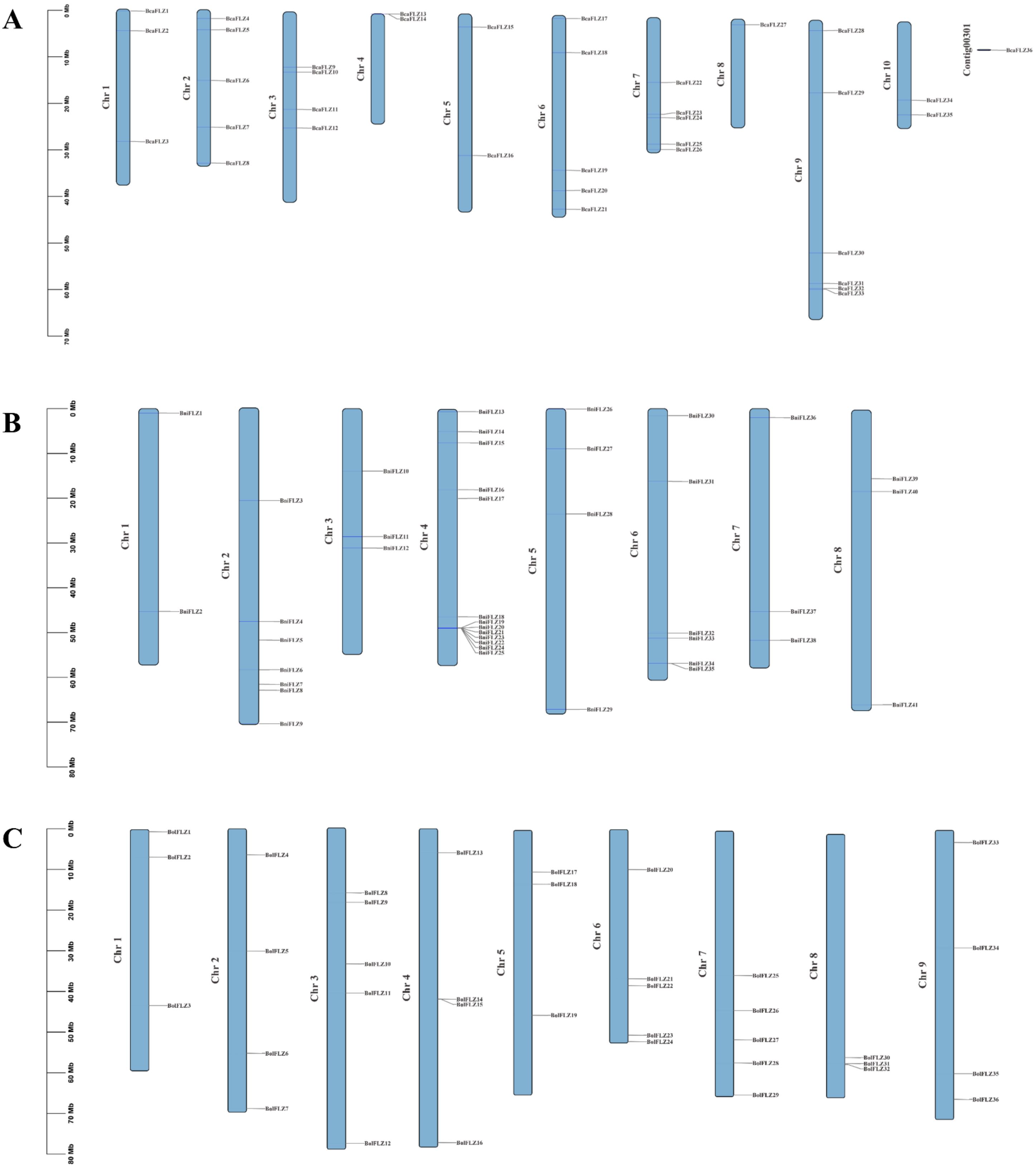

Figure S4 Chromosomal localization of the FLZ gene in three *Brassica* speices. (A) *Brassica rapa* (Bca); (B) *Brassica nigra* (Bni); (C) *Brassica oleracea*; (Bol). Each gene was mapped to the chromosome based on its physical location. The chromosome number is indicated at the left.
